# The Management of Oligoprogression in the Landscape of New Therapies for Metastatic Melanoma

**DOI:** 10.3390/cancers11101559

**Published:** 2019-10-14

**Authors:** Michele Guida, Nicola Bartolomeo, Ivana De Risi, Livia Fucci, Andrea Armenio, Ruggero Filannino, Eustachio Ruggieri, Francesco Macina, Michele Traversa, Annalisa Nardone, Francesco Figliuolo, Federica De Luca, Fabio Mele, Stefania Tommasi, Sabino Strippoli

**Affiliations:** 1 Medical Oncology Department, National Cancer Research Centre “Giovanni Paolo II”, 70124 Bari, Italy; ivana.derisi@gmail.com (I.D.R.); ruggerofi@gmail.com (R.F.); strippoli.sabino@libero.it (S.S.); 2 Department of Biomedical Sciences and Human Oncology, University of Bari, 70124 Bari, Italy; nicola.bartolomeo@uniba.it; 3 Pathology Department National Cancer Research Centre “Giovanni Paolo II”, 70124 Bari, Italy; livia.fucci@gmail.com (L.F.); f.mele@oncologico.bari.it (F.M.); 4 Department of Plastic Surgery, National Cancer Research Centre “Giovanni Paolo II”, 70124 Bari, Italy; andreaarmenio@hotmail.com (A.A.); francescofigliuolo@virgilio.it (F.F.); 5 Department of Surgery, National Cancer Research Centre “Giovanni Paolo II”, 70124 Bari, Italy; eustachio.ruggieri@tiscali.it; 6 Radiotherapy Department, National Cancer Research Centre “Giovanni Paolo II”, 70124 Bari, Italy; f.macina@gmail.com (F.M.); mi.tra@alice.it (M.T.); nardone.annalisa@gmail.com (A.N.); fedluca@yahoo.it (F.D.L.); 7 Molecular Diagnostic and Pharmacogenetics laboratory, National Cancer Research Centre “Giovanni Paolo II”, Bari 70124, Italy; s.tommasi@oncologico.bari.it

**Keywords:** metastatic melanoma, oligoprogression, treatment beyond progression, checkpoint inhibitors, targeted therapy

## Abstract

*Background*: A limited degree of progression after a response to treatment is labelled as oligoprogression and is a hot topic of metastatic melanoma (MM) management. Rogue progressive metastases could benefit from local treatment, which could allow the continuation of ongoing systemic therapy, also known as treatment beyond progression (TBP). *Methods*: We retrospectively reviewed 214 selected MM patients who were treated with v-Raf murine sarcoma viral oncogene homolog B (BRAF)/mitogen-activated-extracellular signal-regulated kinase (MEK) or programmed cell death protein 1 (PD-1) inhibitors and received a local treatment continuing TBP. We performed univariate and multivariable analyses to assess the association between therapy outcomes and a series of clinical and biological features. *Results*: We identified 27 (10%) oligoprogressed patients treated locally with surgery (14), radiosurgery (11), and electrochemotherapy (2). TBP included PD-1 inhibitors (13) and BRAF/MEK inhibitors (14). The median progression-free survival post oligoprogression (PFSPO) was 14 months (5–19 95% confidence interval (C.I.)). In the univariate analysis, a significantly longer PFSPO was associated with complete response (CR), Eastern Cooperative Oncology Group (ECOG) performance status (PS) of 0, neutrophils/lymphocytes ratio (N/L) <2, and progression-free survival (PFS) at oligoprogression >11 months. Nevertheless, in the multivariable analysis, only CR and N/L <2 were found to be associated with longer PFSPO. *Conclusions*: In selected patients, local treatments contribute to controlling oligoprogression for a long time, allowing the continuation of systemic treatment and prolongation of overall survival (OS). Increasing biological and clinical knowledge is improving the accuracy in identifying patients to apply for local ablative therapies.

## 1. Introduction

In metastatic melanoma (MM), the advent of targeted therapies for BRAF-mutated patients and immune checkpoint inhibitors is among the most successful milestones reached in the oncology field, boasting significant improvements in patient survival. However, the therapy paradigm is rapidly evolving, alongside challenging issues that are crowding the space of the oncology debate about the optimal therapeutic strategy in terms of first line choice and sequences [1,2].

These new therapeutic approaches are leading to a careful revision of the traditional criteria used to define the response to therapy as well as the behavior change of melanoma biology over time. In particular, the response evaluation criteria in solid tumors (RECIST) are becoming inadequate to catch the heterogeneity of the progression spectrum, and they miss the definition of a very limited degree of progression after a good response to treatment, labeled as oligoprogression [3,4,5]. This pattern of progression reflects a focal acquired resistance, which stems from intrinsic melanoma clonal heterogeneity added to an extrinsic treatment selective pressure. As such, its biological significance is clearly distinguished from generalized progression due to innate or secondary resistance that involves the majority of disease sites [6,7]. Owing to this distinction, oligoprogression could be managed by applying local treatment, which could control rogue and limited progressive metastases, allowing the maintenance of a still effective systemic therapy [8,9,10,11]. Treatment beyond progression (TBP) has clearly demonstrated advantages in terms of prolonging overall survival in several malignancies, including renal cell carcinoma, breast cancer, non-small cell lung cancer, and colorectal cancer, using different therapeutic approaches such as chemotherapy, immunotherapy, or biological agents [11,12,13,14,15,16,17].

However, despite the biological rational and a huge trend toward local therapies to metastatic disease, there are currently very limited available data on this therapeutic strategy in MM. Although preliminary and heterogeneous clinical experiences are documenting that TBP might be beneficial in some patients treated with either targeted therapy [18,19,20,21,22,23] or immunotherapy [24,25], clear guidelines are lacking. In particular, the previous retrospective reports accounted patients treated beyond progression regardless the degree of progression. Moreover, there are no previous reports about TBP in oligoprogressed patients treated with combination of BRAF and MEK inhibitors as well as there being no data about the role of local treatments in managing oligoprogression during PD1 inhibitors.

Despite the undisputed advantage of TBP in some patients, several uncertainties concerning patient selection, the choice of loco-regional approaches, and disease features are yet to be clarified. In this scenario, the risks of a delay in the adoption of a more effective therapeutic alternative and the exposure to dangerous toxicity, as well as the avoidance of economic costs, should be weighed in the decision-making process.

Therefore, there is a clear need to individualize the treatment management and to identify the subset of patients who could be successfully treated beyond progression and for whom local therapies could share to disease control and to improve survival. In order to outline practical orientation points, we retrospectively reviewed the medical records of MM patients from our institution treated with BRAF/MEK inhibitors and PD1 blockades in order to define the incidence of oligoprogression defined as a condition characterized by a progression of a solitary or few (≤3) metastases in a single site or organ suitable of local treatment, while all the rest of the disease had already responded or was stable to the ongoing systemic therapy. Thus, we evaluate the risk–benefit ratio of TBP combined with various loco-regional therapeutic approaches by building a univariate and multivariable analysis to assess the association between relevant therapeutic outcomes and a series of clinical and biological features, which could predict the optimal patient selection.

## 2. Results

### 2.1. Incidence of Oligoprogression, Clinical Features, and Therapeutic Outcomes of TBP Patients

We retrospectively reviewed the medical records of 214 MM patients corresponding to 278 therapies (109 BRAF/MEK inhibitors and 169 PD1 blockades) performed in a time span between January 2012 and December 2018, 195 cases as first line, and second or further lines for the remaining 83 ones. The cut-off date of the last follow-up was July 2019. We identified 27 patients with oligoprogression, corresponding to an incidence of about 10% of the entire patient population. All this patient population underwent loco-regional therapy on progressive sites.

The main features of our patient population are described in Table 1. Briefly, the features included male/female: 14/13; median age: 56 years (range 35–75 years); median Eastern Cooperative Oncology Group (ECOG) performance status (PS): 0 (range 0–2), with 17 patients having a PS of 0; BRAF status mutated/wild type: 18/9; number of progressed metastases 1/>1 and ≤3: 16/11; LDH at oligoprogression normal/high: 13/14; neutrophils to lymphocytes ratio at oligoprogression >2/<2: 14/13. Sites of oligoprogression included the brain in 10 patients, lymph nodes in eight patients, skin/soft tissue in five patients, bowel in two patients, and liver/gall bladder in two patients. Half of the patients were treated with targeted therapy, and the other half with checkpoint inhibitors.

**Figure 1:**
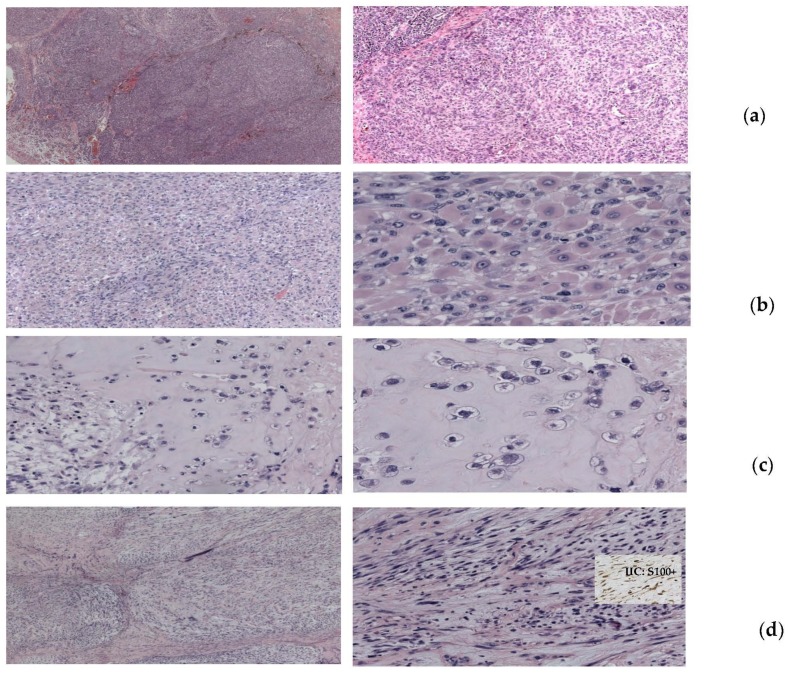
Representative histology of an oligoprogressive lesion of the thoracic wall radically removed with surgery in a patient undergoing anti-BRAF plus anti-MEK targeted therapy. In a pseudolobular pattern with the presence of crammed epithelioid cells of various sizes and hyperchromic nuclei (**a**), numerous aberrant patterns were also present, including rhabdomioid areas with cells of intensely eosinophilic and dense cytoplasm and hypercromic eccentric nuclei (**b**), chondroid areas with cell nests immersed in a cartilaginous-like stroma (**c**), and myxoid areas with spindle cells immersed in abundant clear stroma (**d**). IIC staining showed positivity for S100 protein in all areas.

**Figure 2:**
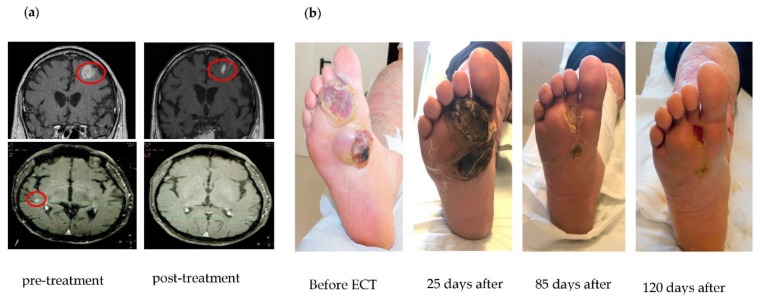
Representative cases of patients treated with different loco-regional treatments on oligoprogression sites while continuing systemic treatments: (**a**) Patient treated with strereotactic surgery on brain metastases and anti-BRAF plus anti-MEK systemic therapy; (**b**) patient treated with local electrochemotherapy and systemic anti-PD-1 immunotherapy.

Local treatments included surgery in 14 patients, radiotherapy in 11 patients, and electrochemotherapy with systemic bleomicin in two patients. All patients obtained a complete response at oligoprogressive sites by these local treatments. Interestingly, a patient undergoing radical surgical removal of a progressive lesion on the thoracic wall, arising during anti-BRAF plus anti-MEK targeted therapy, showed a very heterogeneous and aberrant histology: In a general pseudolobular pattern of crammed epithelioid cells, large rhabdomioid, chondroid, and myxoid areas were also present, while BRAF mutation was maintained in all areas of the lesion (Figure 1). In Figure 2, we report two representative complete responses obtained with strereotactic surgery for brain metastases (Figure 2a) and with electrochemotherapy for cutaneous/subcutaneous oligoprogressed lesions (Figure 2b) arising during targeted therapy and PD-1 checkpoint immunotherapy, respectively.

After radical treatment, all patients continued systemic treatment for at least two months.

**Figure 3:**
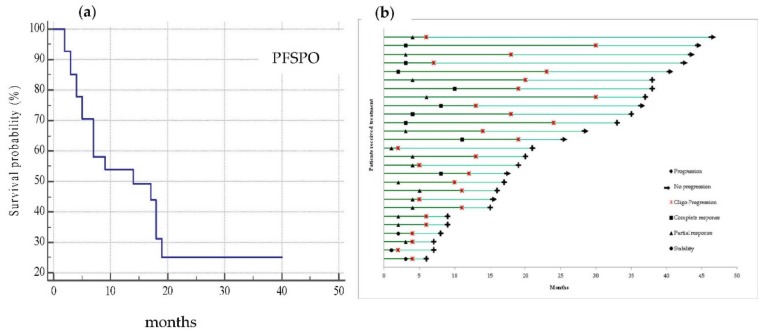
(**a**) Kaplan–Meier curve representative of progression-free survival post oligoprogression (PFSPO) which median value was 14 months (5–19 95% C.I.) with 10 patients not progressed at time of analysis; (**b**) the swimmer plot shows the treatment and follow-up history for all 27 patients who developed an oligoprogression and were treated with local approaches and treatment beyond progression (TBP). Each bar represents one patient.

The median progression-free survival (PFS) at oligoprogression was 18 months (range 7–24 95% confidence interval (C.I.)), while the median progression-free survival post oligoprogression (PFSPO) (which mirrored the contribution of local treatment to disease control) was 14 months (5–19 95% C.I.). Thirty-seven percent of patients had not progressed at the time of analysis (Figure 3a). Before oligoprogression occurred, as shown in the swimmer plot analysis (Figure 3b), the best response was 33% complete response (CR), 56% partial response (PR), and 11% stable disease (SD).

**Figure 4:**
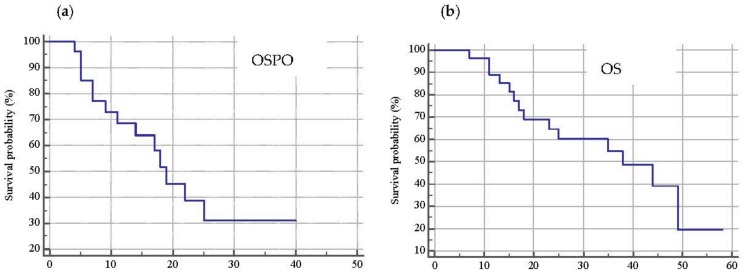
Kaplan–Meier curve representative of overall survival post oligoprogression (OSPO) (**a**) whose median value was 19 months (range 11–25; 95% C.I.) and overall survival (OS) (**b**) with a median value of 38 months (range 18–49; 95% C.I.). At time of analysis, 13 patients were alive.

The median overall survival post oligoprogression (OSPO) was 19 months (range 11–25; 95% C.I.) while the median overall survival (OS) was 38 months (range 18–49; 95% C.I.) with 48% of patients alive at the time of the analysis (Figure 4a,b).

### 2.2. Associations between Therapeutic Outcomes and Clinical and Biological Features

In the univariate analysis, a longer PFSPO was associated with CR (vs. PR, *p* = 0.001, HR 95% C.I. 2.9 (95% C.I. 1.1–7.8)), a ECOG PS of 0 (*p* = 0.0008, HR 95% C.I. 8.5 (2.4–30)), N/L <2 (*p* = 0.03, HR 95% C.I. 3.1 (1.1–8.7)), and the onset of oligoprogression later than 11 months (*p* = 0.04, HR 95% C.I. 2.9 (1.1–7.9)). Multivariable analysis demonstrated that CR (*p* = 0.004, HR 95% C.I. 4.1 (1.5–10.9)) and N/L <2 (*p* = 0.03, HR 95% C.I. 3.1 (1.1–8.8)) remained associated with improved PFSPO.

Regarding survival, in the univariate analysis, a longer OSPO was associated with an ECOG PS of 0 (*p* = 0.0002, HR 95% C.I. 15.5 (3.7–64.0)), a N/L <2 at oligoprogression (*p* = 0.007, HR 95% C.I. 4.8 (1.5–15.3)), the onset of oligoprogression later than 11 months (*p* = 0.03, HR 95% C.I. 3.4 (1.2–10.1)), and the use of surgery (vs. radiotherapy, *p* = 0.04, HR 2.8, 95% C.I. 0.9–8.6). In the multivariable analysis, the only parameter associated with a longer OSPO was an ECOG PS of 0 (*p* = 0.006, HR 95% C.I. 7.3 (1.8–30.3)).

Moreover, OS was longer in patients with CR as best response (vs. PR, *p* = 0.009, HR 95% C.I. 2.6, (0.9–7.6)), an ECOG PS of 0 (*p* = 0.001, HR 95% C.I. 8.5 (2.3–31.3)), LDH < ULN (*p* = 0.01, HR 95% C.I. 4.2 (1.4–12.6)), N/L <2 (*p* = 0.01, HR 95% C.I. 4.2 (1.4–12.8)), onset of oligoprogression later than 11 months (*p* = 0.0007, HR 95% C.I. 80 (2.4–26.2)), skin or lymph node as the site of oligoprogression (vs. visceral metastases, *p* = 0.05, HR 95% C.I. 3.5 (1.1–11.8)), and surgery as local treatment (*p* = 0.02, HR 95% C.I. 3.3 (1.0–10.5)). Also, in the multivariable analysis, a longer OS was associated with an ECOG PS of 0 (*p* = 0.008, HR 95% C.I. 8.5 (1.7–42.0)), LDH < ULN (*p* = 0.01, HR 95% C.I. 0.04 (0.003–0.5)), N/L <2 (*p* = 0.02, HR 95% C.I. 7.1 (1.4–36.6)), onset of oligoprogression later than 11 months (*p* = 0.002, HR 95% C.I. 0.003 (<0.001–0.1)), and surgery as local treatment (*p* = 0.004, HR 95% C.I. 0.3 (0.1–0.7)).

For each outcome, the assumptions of the Cox multivariable model were respected, and the *p*-value for all three overall tests (likelihood, Wald, and score) were significant, indicating that the model was significant. The multivariable analysis is summarized in Table 2.

## 3. Discussion

The aim of our study was to investigate the effect of loco-regional treatment on a selected MM population showing oligoprogression during systemic therapy in order to better characterize which of these patients would benefit from extended systemic treatment.

This therapeutic strategy assumes that ongoing treatment could be still effective in spite of a RECIST-labeled progression. A similar scenario could happen when progression involves limited sites as the stemming of treatment selective pressure or branched cancer evolution after a previous good response to systemic therapy.

In this oligoprogression setting, rogue progressive metastases could be radically cured by loco-regional treatment, leaving only responsive tumor cells. This emerging condition has not yet been extensively investigated in MM, and few data are known about its dimension as well as its management.

In our single-center analysis, we retrospectively analyzed the pattern of progression of 214 MM patients treated with anti-BRAF plus anti-MEK targeted drugs for BRAF-mutated melanoma or with PD1 inhibitors, and we found a 10% incidence of oligoprogression (27 patients).

There are no clear data in the literature about the incidence of this kind of progression among MM patients treated with these drugs, which represent the main actors in melanoma treatment. Indeed, in published retrospective reports concerning experiences of TBP in MM, this therapeutic strategy was adopted in a much higher percentage of patients ranging between 30% and 48% for targeted therapy [20,21,22,23] and between 28% and 51% for checkpoint inhibitor immunotherapy [24,25]. This is why TBP was often offered at the investigator’s discretion regardless of the pattern of progression or the use of loco-regional treatments. Moreover, a clear definition of oligoprogression was not reported [20,21,22,23,24,25].

In our study, we closely selected patients with metastases progressed in a single site and suitable for local treatment. Thus, we collected a homogeneous population who underwent loco-regional approaches achieving a complete response in oligoprogressed sites, while continuing systemic therapy. Thus, our patient population is different and much more homogeneous with respect to those of previous reported experiences.

In our opinion, the management of oligoprogression is one of the keys for success concerning TBP. Of note is that some retrospective analyses reported a more positive impact of TBP when local treatment was offered to patients continuing systemic therapy. In this regard, convincing data on renal cell carcinoma [26,27,28] and melanoma [21] have been reported, despite no data being available regarding the correct local treatment strategies and timing to be used.

We used surgery, radiosurgery, and electrochemotherapy as loco-regional approaches, which allowed the reaching of a complete response on all oligoprogressed sites. This strategy also led us to find a rather long PFSPO of 14 months (5–19 95% C.I.), which was our main interest since this parameter mirrors how local treatment contributes to controlling disease and, consequently, to the patient’s overall survival. Our PFSPO result was much longer than those reported by Puzanov with BRAF inhibitors (3.8 months) or by Long and Beaven, with PD1 inhibitors ranging from 1.4 to 9.4 months [21,24].

Moreover, we observed a median OSPO of 19 months (range 11–25; 95% C.I.) and a median OS of 38 months (16–38 95% C.I.), which are much longer than those reported in previous MM TBP experience both with BRAF inhibitors [22,23], which reported a median OSPO and a median OS ranging from 5.2 to 11.6 and 12.8 to 26 months, respectively, and with PD1 blockade documenting a median OS of 24.4 months [25] and a rate of 24-month survivors of 59% [24].

However, in all the previous reports except for Hassel’s, TBP in MM was associated with a longer median OS compared with no TBP [20,21,22,23,24,25]. Chan and co-workers reported the experience of TBP using the BRAF inhibitors vemurafenib and dabrafenib in 37 of 95 patients accrued within clinical trials, and they showed that known prognostic factors such as ECOG, LDH, and the size of the target lesions were able to influence overall survival from the time of progression, but in multivariable analysis, only TBP was correlated with better survival compared with the cessation of treatment [20]. In a similar retrospective study, Sholtens and co-workers reported the outcomes of 70 patients who experienced progression after prior objective response upon treatment with vemurafenib, and they showed that only stopping treatment at disease progression was significantly associated with shorter survival in the multivariable analysis [22]. Regarding immunotherapy with PD1 inhibitors, both for a pooled, retrospective analysis of data from phase 3 trials of nivolumab by Long [24] and for a pooled analysis from 2624 patients who received anti-PD-1 antibodies pembrolizumab or nivolumab in clinical trials by Beaven [25], an advantage in terms of OS was derived from TBP, which also appeared able to induce a RECIST criteria response >30% at a rate of patients between 14% and 28%. Even so, specific criteria to select which patients should be eligible for TBP at the time of progression were not clearly identified, and both authors emphasize that the patients who benefit from this treatment prolongation are more likely to have multiple prognostic beneficial factors such as lower ECOG, normal LDH, and M1a stage [24,25].

Interestingly, in our population, the presence of a previous CR obtained with systemic therapy and a N/L <2 identified the patients who most benefitted from TBP in terms of PFSPO, both in univariate and multivariate analysis; meanwhile, only in the univariate analysis, an ECOG of 0 and a PFS longer than 11 months were also associated with PFSPO. Furthermore, OSPO was correlated with a good ECOG PS, N/L <2, use of surgery as local treatment and PFS from beginning of treatment >11 months. Nevertheless, in the multivariable analysis, an ECOG PS of 0 resulted as the only parameter to correlate with a longer OSPO.

We also found a close correlation of OS with CR as best response, an ECOG PS of 0, normal LDH at oligoprogression, N/L <2, PFS from start of therapy >11 months, skin or lymph node as the site of oligoprogression vs. visceral sites, and surgery as local treatment. In the multivariate analysis, ECOG PS of 0, normal LDH, N/L <2, PFS from start of therapy >11 months, and surgery as local treatment maintained their significant role.

Intriguingly, regardless of the type of systemic therapy (targeted or immunological), we underline the relevant role of the N/L ratio evaluated at the time of oligoprogression both in PFSPO and OSPO. Basal neutrophils and N/L ratio have been recently reported to be prognostic in patients with MM receiving ipilimumab anti-CTLA4 [29] or nivolumab anti-PD-1 [30]. Even if the role of this hematological index could mirror the immune response and is never investigated in patients treated with BRAF/MEK inhibitor, as is already known, targeted therapy is also involved in immune surveillance and immune response acting, both at the level of the neoplastic cell and the microenvironment [31].

Furthermore, CR is a well-known positive prognostic factor, both for targeted therapy and immunotherapy and is able to impact significantly on PFS and OS [32,33].

Regarding local therapy, as previous mentioned, only surgery showed a correlation with OS. In addition to treatment, surgery allows to show the histological and biological characteristics of progressive sites. This was the case in one of our patients, who showed a very aberrant histological heterogeneity that became resistant to anti-BRAF/anti-Mek despite the fact that the BRAF mutation was maintained (Figure 1).

Despite our positive results, there are some limitations to this study. First of all, its retrospective design increases the risk of incurring bias in patient selection and analysis. Secondly, the small number of patients included might give a distorted estimation of patients’ characteristics and results. Moreover, we used clinical and biological parameters, which are mainly prognostic and not predictive of drug specific outcomes. Finally, it is necessary to consider the empiric choice of the different loco-regional approaches used. Thus, the standardization of loco-regional techniques needs to be better clarified in matched prospective patient populations.

In this direction, prospective studies, such as the ongoing NCT03514901 with BRAF and MEK inhibitors, could help to identify and validate specific predictive factors to better select patients who could obtain long-term disease control with TBP. Future directions should yield serological tools able to provide real-time changing of the degree of clonal heterogeneity of melanoma. These minimal invasive biomarkers could uncover the pharmacodynamic useful of a TBP by mirroring how true is the extent of oligoprogression.

Despite these limitations and the absence of published guidelines regarding this topic, the present study confirms that TBP after loco-regional treatment on oligoprogressing sites seems to be associated with a prolongation of both disease control and OS, allowing clinicians to have further therapeutic choices in case of progression. Thus, this option should be considered in selected cases to offer the right treatment to the right patient at the right time.

## 4. Materials and Methods

We conducted a single-center, observational cohort study by retrospectively recruiting patients with stage IV melanoma treated with BRAF/MEK inhibitors, for BRAF-mutated patients, or PD-1 inhibitors. All patients included in this analysis were treated beyond oligoprogression for at least two months and received local treatment on rogue progressive metastases. Systemic therapy included targeted therapy (dabrafenib and trametinib or vemurafenib and cobimetinib), or PD-1 inhibitors (pembrolizumab or nivolumab) according to standard therapeutic doses and schedules. We first analyzed the pattern of progression and defined as oligoprogression a condition characterized by a progression of a solitary or few (≤3) metastases in a single site or organ, while all the rest of the disease had already responded or was stable to the ongoing systemic therapy. Conversely, patients with generalized progression were excluded. Additional eligibility criteria included measurable disease as per the response evaluation criteria in solid tumors (RECIST) criteria and an Eastern Cooperative Oncology Group (ECOG) performance status (PS) ≤2. For each patient, we collected histological, biological, and clinical features and the type of loco-regional treatment.

First of all, we calculated the incidence of oligoprogression among the entire patient population. The primary evaluated therapeutic outcome was the progression-free survival post oligoprogression (PFSPO), defined as the time interval between the date of oligoprogression radically treated with loco-regional therapy and the date of subsequent clinical or radiological progression. The main secondary outcomes included: 1. Overall survival post oligoprogression (OSPO), defined as the time interval between radically treated oligoprogression and the date of death or of the last follow-up; 2. overall survival (OS), defined as the time interval between the date of the beginning of the current systemic therapy and the date of death or of the last follow-up.

In our analysis, we focused on PFSPO as the therapeutic outcome, since this parameter is a direct expression of the contribution of loco-regional treatments to the control of disease and, consequently, to the patient’s overall survival.

Overall survival and progression-free survival were calculated with the Kaplan–Meier method and expressed as medians and their 95% confidence interval (C.I.).

We performed a univariate analysis to define the associations between PFSPO, OSPO, and OS with some clinical and biological features, which included age, sex, kind of melanoma, BRAF status, type of systemic therapy, line of therapy, best response before oligoprogression, M stage and site of metastases before start of treatment, site of oligoprogression, local treatment adopted, ECOG PS, as well as LDH, number of white blood cells (WBC), and, among these, the number of neutrophils, lymphocytes, and their ratio. These last hematological parameters and ECOG PS were recorded when oligoprogression occurred. A log-rank test was used for the comparison between the survival curves, and the hazard ratio with 95% C.I. was also calculated to compare the hazard in two groups.

Variables with *p*-values lower than 0.05 in the univariate analysis were included in multivariable Cox-regression models. Stepwise selection, using *p*-value < 0.05 as the entry criterion and *p*-value ≥ 0.15 as the removal criterion, was used to estimate the final models. The proportional hazard assumptions for the Cox model were checked, and three overall tests (likelihood, Wald, and score) were performed in order to evaluate the significance of the final models.

All tests of statistical significance were two-tailed, and *p*-values less than 0.05 were considered statistically significant. Statistical analysis was performed in R 3.5.3 software using the “RcmdrPlugin.EZR” package.

## 5. Conclusions

The management of oligoprogression is one of the hot topics regarding melanoma patients undergoing systemic treatments beyond disease progression. The correct management of oligoprogression could support the potential benefit of continued TBP.

In our experience, oligoprogression, defined as appearance of new lesions, which is confined in single site and is amenable of eradication, is a rare event arising in about 10% of patients who have previously obtained a good response to systemic treatments for MM. Loco-regional treatments allowed us to control all metastatic sites for quite a long time, with a prolongation of the median post-oligoprogression progression-free survival and overall survival.

Owing to the absence of shared rules and robust literature data, there is a clear need of increased biological and clinical knowledge to improve accuracy in identifying patients to apply for local ablative therapies, contributing to the generation of guidelines for their appropriate use. Although the limitations of our retrospective analysis, we reasoned that two main features like a previous complete response and a simple blood derived parameter such as the ratio of the neutrophil and lymphocyte count could provide strong indications to offer this therapeutic strategy. Thus, the mirage of TBP and oligometastasis management is becoming a treatment paradigm, although prospective trials will be needed to identify and validate specific predictive factors for selecting patients and to standardize the different local treatment modalities.

An optimal patient management is required by clinicians with great expertise and capable of proposing the right treatment for the right patient at the right time.

## Figures and Tables

**Table 1:** Main patient features.

Features	% (n)
Median age (range)	56 (35–75) years
Male	52 (14)
Female	48 (13)
Type of melanoma	
Cutaneous	85 (23)
Unknown origin	15 (4)
Molecular status	
BRAF V600	67 (18)
NRAS Q61	7 (2)
Wild type	26 (7)
DFS median (range)	16 (0–360) months
M stage	
M1a	33 (9)
M1b	26 (7)
M1c	15 (4)
M1d	26 (7)
Systemic therapy	
PD1 inhibitors	48 (13)
Targeted therapy	52 (14)
Line of therapy	
First line	41 (11)
Second line	33 (9)
Third line	26 (7)
Best response	
Complete response	33 (9)
Partial response	56 (15)
Stable disease	11 (3)
Sites of oligoprogression	
Skin	19 (5)
Lymph nodes	30 (8)
Liver and gallbladder	7 (2)
Bowel	7 (2)
Brain	37 (10)
Number of progressed metastases	
1	59(16)
2	26 (7)
3	15(4)
Local therapy	
Surgery	52 (14)
Radiotherapy	41 (11)
Electrochemotherapy	7 (2)
LDH at oligoprogression	
Under the upper limits of normal	48 (13)
Over the upper limits of normal	52 (14)
Eastern Cooperative Oncology Group (ECOG) performance status (PS)	
0	63 (17)
1	33 (9)
2	4 (1)
Neutrophils to lymphocytes ratio	
<2	48 (13)
>2<3	30 (8)
>3	22 (6)

**Table 2:** Multivariable analysis of clinical and biological features that correlate with OS, PFSPO, and OSPO.

Features	OS			PFSPO			OSPO		
	HR	95% C.I.	P	HR	95% C.I.	P	HR	95% C.I.	P
Neutrophils/lymphocytes (N/L) ratio (<2 vs. >2)	7.15	1.40–36.61	0.018	3.10	1.09–8.81	0.034	3.93	0.95–16.21	0.058
Best response (complete response (CR) vs. others)	NI^+^	NI	NI	4.11	1.55–10.88	0.004	NI	NI	NI
ECOG PS (0 vs. others)	8.54	1.74–42.01	0.008	NI	NI	NI	7.31	1.76–30.35	0.006
Local approches (surgery vs. others)	0.28	0.11–0.67	0.004	NI	NI	NI	0.59	0.34–1.03	0.066
LDH < ULN	0.04	0.003–0.47	0.010	NI	NI	NI	NI	NI	NI
Progression-free survival (PFS) >11 months	0.003	<0.001–0.12	0.002	NI	NI	NI	NI	NI	NI

NI^+^: Not included.
